# Mandarin-Speaking Children’s Speech Recognition: Developmental Changes in the Influences of Semantic Context and *F*_0_ Contours

**DOI:** 10.3389/fpsyg.2017.01090

**Published:** 2017-06-28

**Authors:** Hong Zhou, Yu Li, Meng Liang, Connie Qun Guan, Linjun Zhang, Hua Shu, Yang Zhang

**Affiliations:** ^1^International Cultural Exchange School, Shanghai University of Finance and EconomicsShanghai, China; ^2^Department of Cognitive Science and ARC Centre of Excellence in Cognition and Its Disorders, Macquarie University, SydneyNSW, Australia; ^3^College of Allied Health Sciences, Beijing Language and Culture UniversityBeijing, China; ^4^School of Foreign Studies, University of Science and Technology BeijingBeijing, China; ^5^National Key Laboratory of Cognitive Neuroscience and Learning, Beijing Normal UniversityBeijing, China; ^6^Department of Speech-Language-Hearing Sciences and Center for Neurobehavioral Development, University of Minnesota, MinneapolisMN, United States

**Keywords:** semantic context, fundamental frequency contours, speech recognition, interfering speech, children

## Abstract

The goal of this developmental speech perception study was to assess whether and how age group modulated the influences of high-level semantic context and low-level fundamental frequency (*F*_0_) contours on the recognition of Mandarin speech by elementary and middle-school-aged children in quiet and interference backgrounds. The results revealed different patterns for semantic and *F*_0_ information. One the one hand, age group modulated significantly the use of *F*_0_ contours, indicating that elementary school children relied more on natural *F*_0_ contours than middle school children during Mandarin speech recognition. On the other hand, there was no significant modulation effect of age group on semantic context, indicating that children of both age groups used semantic context to assist speech recognition to a similar extent. Furthermore, the significant modulation effect of age group on the interaction between *F*_0_ contours and semantic context revealed that younger children could not make better use of semantic context in recognizing speech with flat *F*_0_ contours compared with natural *F*_0_ contours, while older children could benefit from semantic context even when natural *F*_0_ contours were altered, thus confirming the important role of *F*_0_ contours in Mandarin speech recognition by elementary school children. The developmental changes in the effects of high-level semantic and low-level *F*_0_ information on speech recognition might reflect the differences in auditory and cognitive resources associated with processing of the two types of information in speech perception.

## Introduction

Speech recognition involves smooth and rapid integration of perceptual and cognitive systems, during which lower- and higher-level information sources in the spoken language are analyzed and synthesized (Moore et al., 2008). In adverse listening conditions, acoustic-phonetic cues are often insufficient for the identification of particular words because of speech signal quality (e.g., distortion to speech sounds caused by poor articulation) or listening background (e.g., the presence of noise or interfering speech). In such circumstances, various supportive mechanisms, especially the semantic context from the preceding words in an utterance or discourse can help the listener perceive, recognize, and understand subsequent words. Previous studies have shown that these factors including quality of speech signal, listening background, and semantic context interact with each other in jointly affecting speech recognition outcome (Pichora-Fuller et al., 1995; Sheldon et al., 2008; Benichov et al., 2012). For example, Laures and Bunton (2003) found that acoustically degraded sentences with flattened fundamental frequency (the lowest frequency of a periodic complex sound and is the acoustic correlate of pitch for speech and music, hereafter referred to as *F*_0_) contours were as intelligible as original sentences with natural *F*_0_ contours when both sentence types were presented in quiet. However, the flattened *F*_0_ contours yielded decreased speech intelligibility in white noise and multi-talker babble backgrounds, indicating the significant interaction between the quality of speech signal and listening background during speech recognition. Dubno et al. (2000) showed that both older and young adults derived greater contextual benefit for recognizing the sentence-final word in the presence of masking broadband noise when compared with quiet, indicating the significant interaction between listening condition and semantic context for speech intelligibility.

The task of listening to speech under suboptimal circumstances is challenging for listeners of all ages, but the detrimental effects of degraded speech and auditory masking appear to be particularly large during childhood because children are not born with fully mature auditory processing capabilities but instead demonstrate a developmental time course of improvement in processing complex auditory situations (Eisenberg et al., 2000; Hall et al., 2002; Blandy and Lutman, 2005; Stuart, 2005; Wilson et al., 2010). Children’s difficulty in perceiving speech in noise and against interfering speech has been investigated in a number of previous studies, which have consistently shown that children need more favorable conditions than adults to achieve comparable speech-in-noise scores (Papso and Blood, 1989; Fallon et al., 2000; Stuart et al., 2006). For example, Fallon et al. (2000) examined sentence recognition by young adults and children of three age groups (5, 9, and 11 years) in the presence of an eight-talker babble and found that children of all the three groups required a more favorable signal-to-noise ratio (SNR) to perform as well as the young adults. This child–adult difference was largest for the youngest children, reflecting the developmental progress in the ability to recognize speech in multi-talker babbles. Stuart (2005) suggested a similar disadvantage for children in speech recognition in noise when comparing the performances of 6- to 15-year-old children and young adults on the NU-CHIPS (the Northwestern University Children’s Perception of Speech test) in quiet, steady-state and interrupted background noise. The younger children exhibited greater difficulty for listening in both kinds of background noise than the older children and adult-like performance was only exhibited in the children 11 years of age and older.

Previous work suggests that younger children are able to use semantic context to assist speech recognition in quiet as early as 2-years-old involving predictive coding to recognize the sentence-final nouns (Fernald, 2001). The ability to use semantic context in adverse listening backgrounds continues to improve into adolescence with significant interactions among the factors of listening background, semantic context and age. For example, investigation of speech intelligibility of high- and low-predictability sentences in children at 9–17 years of age showed that contextual cues assisted children of all ages in identifying words masked by babbles with older children performing better than younger children in the high context conditions (Elliot, 1979). Similar studies including children aged 5–11 years (Fallon et al., 2000) or 5–10 years (Stelmachowicz et al., 2000) also supported age-related improvement in contextual benefit for recognizing speech in adverse listening backgrounds.

In addition to the line of work on the effects of adverse listening backgrounds, there has been another line of investigation on the effect of degraded speech signal on speech recognition by children. Smiljanic and Sladen (2013) found that clear speech with more pauses, longer sentence/vowel durations and greater vowel spaces was more intelligible than conversational speech for children aged 6–13 years. In addition, children have the ability to use semantic context to decode degraded signals in quiet. For example, in a quiet listening condition, Cole and Perfetti (1980) found that 4-years-old were better at detecting mispronounced words in high-predictability sentences than in low-predictability sentences. Craig et al. (1993) found that children 8–10 years of age required shorter portions of the target words (i.e., less acoustic-phonetic information) for recognition in high-predictability than in low-predictability sentences. By contrast, children aged 5–7 years required as much acoustic-phonetic information with high-predictability as with low-predictability contexts to identify target words.

To our knowledge, previous studies have not combined the two lines of work to investigate whether children can use sentence context to aid recognition of degraded speech in adverse listening backgrounds. There is also a lack of studies in which the primary target language is not English to test the generalizability of previous findings on other languages with typologically distinct acoustic and prosodic properties. In a tonal language like Mandarin Chinese, *F*_0_ is primarily used to distinguish lexical meanings (Wang, 1973). This differs greatly from non-tonal languages (e.g., English and French) in which *F*_0_ information is mainly used for pragmatic purposes such as sentence modality (interrogative vs. declarative), emphasis and emotion (Cutler et al., 1997). Although lexical tones have a phonemic role in Mandarin, alteration of the original *F*_0_ contours has little effect on the intelligibility of sentences in quiet. In contrast, adverse listening conditions (e.g., interfering speech) substantially reduce the intelligibility of Mandarin sentences with altered *F*_0_ contours compared with sentences with natural *F*_0_ patterns (Wang et al., 2013; Chen et al., 2014). Developmental research reveals that Mandarin-speaking children show sensitivity to *F*_0_ information several days after birth (Wermke et al., 2016) and acquire the *F*_0_ patterns of lexical tones in perception and production at an early age (Hua and Dodd, 2000). Considering the interaction of sentential semantic context, *F*_0_ contours and listening condition on Mandarin speech recognition by young adults (Wang et al., 2013), it remains unclear how these factors may separately or jointly affect a child’s performance.

The purpose of the present study aimed to investigate the possible different effects of high-level semantic and low-level *F*_0_ information on the recognition of Mandarin speech by elementary and middle school children in quiet and single-talker interference conditions. On the basis of previous studies that have suggested a developmental/maturational course for children’s ability to recognize speech in adverse listening conditions (Elliot, 1979; Stelmachowicz et al., 2000; Stuart, 2005), we predicted that the middle school-aged children would perform better than the elementary school children in the presence of interfering speech, while the two groups would perform equally well in quiet. Furthermore, the middle school-aged children would benefit more from sentence context than the elementary school children in the interfering background but not in quiet. More importantly, we expected a significant interaction between age group and *F*_0_ contours. Specifically, the middle school children would perform better than the elementary school children when *F*_0_ contours were flattened, while both groups would perform similarly on recognizing speech with natural *F*_0_ contours, assuming that younger children might rely more on natural *F*_0_ patterns than older children in speech recognition. The effect of age group on *F*_0_ contours might be further modulated by listening background and semantic context, e.g., the middle school children could benefit more from semantic context than the elementary school children when *F*_0_ patterns changed from natural contours to flat contours.

## Materials and Methods

### Subjects

Forty-eight elementary school children (23 boys, Grade 4, age range 9.6–10.1 years) and forty-six middle school children (25 boys, Grade 8, age range 13.5–14.2 years) from local schools in Beijing were recruited. All children were native speakers of Mandarin Chinese and none of the children had a history of any neurological or psychiatric disorders. Audiometric evaluation was carried out using a GSI 61 clinical audiometer (Grason-Stadler, Inc., Madison, WI, United States) with a standard Hughson-Westlake approach and each child had pure-tone thresholds no greater than 20 dB HL at octave intervals from 250 to 8000 Hz bilaterally. Written informed consent was obtained from the children and their legal guardians. The study was approved by the Institutional Review Boards (IRBs) at Beijing Language and Culture University and Beijing Normal University, and was conducted in compliance with the Declaration of Helsinki.

This study focused on the two age groups because children 11 years of age and older exhibited adult-like performance on speech recognition in noise (Stuart, 2005). A mixed-design was adopted with semantic context (normal sentence vs. word list sentence) and *F*_0_ contours (normal vs. flat) as within-subject factors, while listening background (quiet vs. interference) and age group (elementary school vs. middle school) as between-subject factors. The participants (boys/girls) randomly divided in the 4 between-subject conditions were distributed as follows: elementary school/quiet, number of subjects (*n*) = 24 including 11 boys and 13 girls; elementary school/interference background, *n* = 24 including 12 boys and 12 girls; middle school/quiet, *n* = 23 including 13 boys and 10 girls; middle school/interference background, *n* = 23 including 12 boys and 11 girls.

### Stimuli

**FIGURE 1:**
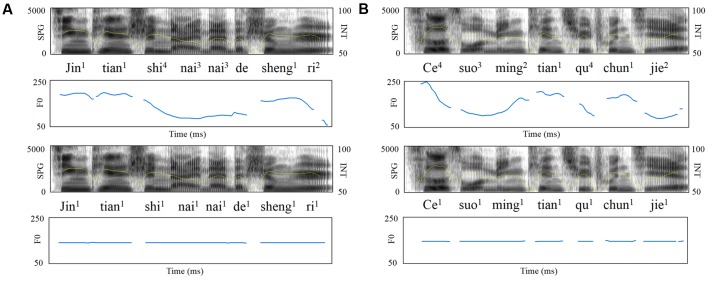
Acoustic features of sample speech stimuli. Broadband spectrograms (SPG: 0–5 kHz), intensity envelopes (INT: 50–100 dB), and fundamental frequency contours (F0: 0–300 Hz) are displayed for **(A)** normal sentence and its pitch-flattened counterpart; **(B)** word list sentence and its pitch-flattened counterpart.

Four types of sentences were used as targets. Specifically, the target sentences included normal/word list sentences with natural/flat *F*_0_ contours, respectively, manipulating semantic context and *F*_0_ contours. Such manipulations have been adopted in our previous studies (Wang et al., 2013; Zhang et al., 2016; Jiang et al., 2017). The normal sentences were 28 declarative Chinese sentences with each sentence comprised of 3 to 6 words (2–4 content words plus 0–2 functional words) that were familiar to both the elementary and middle school children. Words from the entire pool of the normal sentences were pseudo-randomly selected to form the word list sentences matched in number of content and functional words with the normal sentences. Word list sentences were syntactically correct but semantically meaningless at the whole sentence level. That is, recognition of both word list and normal sentence recruits syntactic and semantic processing, but only normal sentence provides the meaningful semantic context of “who does what to whom” beyond random lexical semantics. A male native Chinese speaker read the normal and word list sentences. Monotonous sentences with flat *F*_0_ contours were created by flattening the natural *F*_0_ contours at each sentence’s mean *F*_0_ (**Figure 1**).

Masker stimuli were consonant-misplaced sentences, which were constructed based on the normal sentences. Specifically, the initial consonant of every syllable in each normal sentence was replaced with another consonant as long as the phonotactic rules of Mandarin were not violated. Consonant-misplaced sentences were unintelligible at both word and sentence levels, thus having minimal effect of informational masking (Wang et al., 2013; Xu et al., 2013). A female native Mandarin speaker read the masker sentences, which was to enable separation of the target message from the interfering speech. Without this indexical information, participants would have to be trained on the identity of the target speaker. The listeners were instructed to listen to the male speaker for the content rather than the identity of the target speaker throughout (Scott et al., 2004). The target and masker sentences were first edited to be at the sound pressure levels of 75 and 70 dB, respectively, and then combined to form the speech against interference stimuli with SNR level set at +5 dB.

### Procedures

We followed similar experimental procedures in our previous studies (Wang et al., 2013; Zhang et al., 2016), except that listeners were instructed to verbally repeat rather than write down the whole target normal/word list sentences in order to reduce cognitive burden on the children. Their responses were recorded and scored by the first author of this paper and checked by an independent auditor blind to the experiment. A strict score standard was adopted. Specifically, only words with consonants, vowels and lexical tones all correctly identified were considered correct answers. During the experiment, the listeners were seated in a sufficiently silent room (not higher than 15 dB for ambient noise level). A set of Edifier R18 loudspeakers were used to present the stimuli with the sound level set at 65 dB SPL calibrated at the listeners’ head. Because *F*_0_ contours and semantic context were within-subject factors, each listener was presented with a total of 56 trials—14 normal sentences and 14 word list sentences with natural or flat *F*_0_ contours. Each child listened to half of the total normal sentences/wordlist sentences (14/28) with natural *F*_0_ contours and the other half with flat *F*_0_ contours, i.e., each child did not listen to the same normal sentence/wordlist sentence with natural or flat *F*_0_ contours. *F*_0_ patterns were counterbalanced across subjects. The experiment adopted self-paced paradigm and the children were encouraged to guess which words they heard. Each normal/word list sentence was used only once with each subject. Before the actual experiment, all children participated in a brief practice session representing samples of all conditions.

## Results

A keyword-correct count was used to calculate the speech recognition sore, i.e., percentage of content words correctly identified (Scott et al., 2004; Wang et al., 2013) (**Table 1**). In order to test the main and interaction effects, a repeated measures ANOVA was conducted with listening condition and age group as between-subject factors, and sentence context and *F*_0_ contours as within-subject factors. Results revealed significant main effects of all the four factors [semantic context: *F*(1,90) = 275.877, *p* < 0.001, $\eta_{p}^{2}$ = 0.754; listening background: *F*(1,90) = 112.168, *p* < 0.001, $\eta_{p}^{2}$ = 0.555; *F*_0_ contours: *F*(1,90) = 384.19, *p* < 0.001, $\eta_{p}^{2}$ = 0.81; age group, *F*(1,90) = 32.236, *p* < 0.001, $\eta_{p}^{2}$ = 0.264], and four significant two-way interaction effects as well [interaction between semantic context and listening condition: *F*(1,90) = 12.53, *p* < 0.001, $\eta_{p}^{2}$ = 0.122; interaction between *F*_0_ contours and age group: *F*(1,90) = 22.464, *p* < 0.001, $\eta_{p}^{2}$ = 0.2; interaction between listening background and *F*_0_ contours: *F*(1,90) = 32.48, *p* < 0.001, $\eta_{p}^{2}$ = 0.265; and interaction between *F*_0_ contours and semantic context: *F*(1, 90) = 4.866, *p* = 0.03, $\eta_{p}^{2}$ = 0.051].

**Table 1:** Mean accuracy (±standard deviation) of each condition for both groups.

	Natural *F*_0_		Flat *F*_0_	
		
	Normal sentence	Word list sentence	Normal sentence	Word list sentence
		**Elementary school children**				
	
Quiet	0.94 (±0.05)	0.84 (±0.08)	0.82 (±0.12)	0.66 (±0.13)
+5 dB SNR	0.83 (±0.12)	0.63 (±0.12)	0.54 (±0.14)	0.39 (±0.16)

		**Middle school children**				
	
Quiet	0.97 (±0.05)	0.90 (±0.05)	0.92 (±0.04)	0.77 (±0.10)
+ 5 dB SNR	0.89 (±0.08)	0.72 (±0.13)	0.75 (±0.14)	0.54 (±0.13)


**FIGURE 2:**
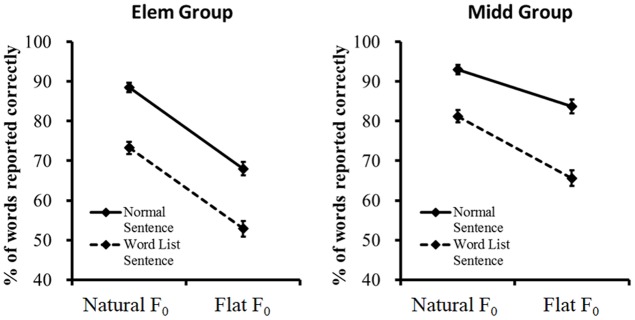
Word-report scores of the simple interaction effects conducted on the significant 3-way interaction between age group, *F*_0_ contours and semantic context. Error bars represent standard deviation across subjects.

There were also two significant 3-way interaction effects [interaction between age group, *F*_0_ contours and semantic context: *F*(1,90) = 5.271, *p* = 0.024, $\eta_{p}^{2}$ = 0.055, and interaction between *F*_0_ contours, semantic context and listening background: *F*(1,90) = 7.292, *p* = 0.008, $\eta_{p}^{2}$ = 0.075]. The four-way interaction effect was not significant [*F*(1,90) = 1.155, *p* = 0.285, $\eta_{p}^{2}$ = 0.013]. Considering the primary purpose of the present study was to investigate the developmental difference between elementary and middle school children in the use of semantic context and *F*_0_ contours during Mandarin speech recognition, the following analyses focused on the significant 3-way interaction between age group, *F*_0_ contours and semantic context. Specifically, this interaction effect was decomposed step by step (Maxwell and Delaney, 2004). First, separate two-way ANOVAs were carried out to examine the simple interaction effects. Results showed that significant interaction between *F*_0_ contours and semantic context was observed in the middle school children [*F*(1,45) = 7.837, *p* = 0.008, $\eta_{p}^{2}$ = 0.151], but absent in the elementary school children [*F*(1,47) = 0.006, *p* = 0.941, $\eta_{p}^{2}$ < 0.001] (**Figure 2**). Second, follow-up analyses were carried out on the simple interaction effects to examine the simple main effects. Results showed that for both the elementary and middle school children, normal/word list sentence with natural *F*_0_ contours were better recognized than their counterparts with flat *F*_0_ contours; normal sentence with natural/flat *F*_0_ contours were better recognized than their word list counterparts [elementary school children: *F*(1,47) > 63.096, *p* < 0.001, $\eta_{p}^{2}$ ≥ 0.573; middle school children: *F*(1,45) > 32.801, *p* < 0.001, $\eta_{p}^{2}$ ≥ 0.422].

## Discussion

The present developmental study explored Mandarin speech recognition by children beyond the age of 9. Two distinct patterns emerged in assessing whether and how age group modulate the influences of low-level *F*_0_ contours and high-level semantic context on Mandarin speech recognition in quiet and interference backgrounds. Specifically, there was a significant modulation effect of age group on *F*_0_ contours, indicating that the elementary school children relied more on natural *F*_0_ contours than the middle school children during Mandarin speech recognition. By contrast, there was no significant modulation effect of age group on semantic context, indicating that children of both age groups used semantic context to assist speech recognition to a similar extent. Furthermore, the significant modulation effect of age group on the interaction between *F*_0_ contours and semantic context revealed that younger children could not make better use of semantic context in recognizing speech with flat *F*_0_ contours compared with natural *F*_0_ contours, while older children benefited more from semantic context when natural *F*_0_ contours were altered, thus confirming the important role of *F*_0_ contours in Mandarin speech recognition by the elementary school children.

Children’s ability to recognize speech in suboptimal listening backgrounds improves with age (Elliot, 1979; Stuart, 2005), and their ability to use semantic context to assist speech recognition also shows a developmental change from childhood to adolescence (Fallon et al., 2000; Stelmachowicz et al., 2000). For the present study that focused on elementary and middle school-aged children, our results showed that both groups of children performed better on speech recognition in quiet than in the interference background and that both groups benefited from semantic context to a similar degree regardless of listening backgrounds. These results indicate that Mandarin-speaking children 9.6–10.1 of age (Grade 4) have developed the ability to recognize speech against an interfering talker as well as the ability to use semantic context to assist speech recognition, both of which are comparable with those of children 13.5–14.2 of age (Grade 8). Because only two age groups of children were included in this study, it is unclear at what age children start to develop such abilities comparable with the middle school-aged children.

Although recognition of degraded speech by children has been investigated in several previous studies (e.g., Smiljanic and Sladen, 2013), how alterations in natural *F*_0_ contours affect speech intelligibility for children has rarely been investigated. Natural *F*_0_ contours play an important role in speech recognition because dynamic changes in natural *F*_0_ contours help listeners recognize voice/unvoiced phonetic segments, separate words in continuous speech and focus attention on the content words. Infants are sensitive to *F*_0_ patterns of speech. For example, newborns perceive emotion conveyed via *F*_0_ contours in maternal speech (Fernald and Simon, 1984) and distinguish between languages based on prosodic information (Nazzi et al., 1998; Ramus et al., 2000). Even newborns’ cry melody is influenced by *F*_0_ patterns of their native languages (Mampe et al., 2009). Our finding that recognition of speech with flat *F*_0_ contours degraded to a greater extent in the elementary school children than in the middle school children indicate that younger children rely more on natural *F*_0_ contours to assist speech recognition. One plausible explanation is that flattening natural *F*_0_ contours alters the acoustic-phonetic features of some speech segments and contrasts between words and makes it more difficult for younger children to parse continuous speech into meaningful units, while older children are likely to have developed highly effective and efficient strategies for compensating for the detrimental effects by focusing their attention on the cues that are less vulnerable to *F*_0_ distortion. An alternative explanation is that the monotonous speech with flat *F*_0_ contours are not attractive enough to the younger listeners, thus resulting in additional costs related to attentional abatement when compared with the older children.

Previous work on young adults shows that *F*_0_ contours affect speech recognition interactively with listening background and semantic context. In particular, flattening or inverting *F*_0_ contours lowers speech intelligibility to a larger degree in adverse listening backgrounds than in quiet no matter whether the target language is a non-tonal or tonal language; the detrimental effect, however, is more serious for a tonal language (Binns and Culling, 2007; Miller et al., 2010; Patel et al., 2010). In addition, alteration in *F*_0_ contours deteriorates speech intelligibility of low-predictability/word list sentences more than high-predictability/normal sentences (Wang et al., 2013; Chen et al., 2014). In the present study, we found a significant modulation effect of age on the interaction between *F*_0_ contours and semantic context, but the interaction between *F*_0_ contours and listening background was not modulated by age. Specifically, for the elementary school children, semantic context did not provide extra benefit when *F*_0_ contours were flattened compared with speech with natural *F*_0_ contours. However, for the middle school children, semantic context did provide extra benefit when *F*_0_ contours were flattened. These results seem to indicate that the middle school-aged children could use semantic context to compensate for the degraded speech signal caused by flat *F*_0_ contours, while this ability is not well developed in the elementary school children. By contrast, both age groups of children benefited to a similar extent from semantic context when the listening background changes from quiet to interference condition, which indicates that the elementary and middle school children are capable of using semantic context to resist interfering speech. Speech recognition by young and older adults in quiet and single-talker interference backgrounds has been explored in our previous studies (Wang et al., 2013 for young adults, and Jiang et al., 2017 for older listeners). Although it is unjustifiable to make a direct statistical comparison across the results of different age groups because different word materials and elicitation approaches were adopted, it is meaningful to compare the different patterns of interaction effects between semantic context and *F*_0_ contours/listening background across the four age groups. For the middle school children and young adults, interaction effects between semantic context and *F*_0_ contours/listening background are both significant, indicating that the two groups could use semantic context to compensate for the distorted speech signal caused by flat *F*_0_ contours and interfering speech. Interestingly, for the elementary school children, only the interaction effect between semantic context and listening background is significant; while for the older listeners, only interaction effect between semantic context and *F*_0_ contours is significant. The modulation effects of age on the interactions between semantic context and *F*_0_ contours/listening background are likely to be related to the maturational/decaying process of speech recognition with degraded signal. Our data indicate that the different age groups may have different receptive skills in processing distortions caused by competing speech and flattened *F*_0_ contours. Competing speech results in acoustic and informational masking to the recognition task, while altering *F*_0_ contours distorts the target speech signal. That is, competing speech and flattened *F*_0_ contours introduces external and internal distortions, respectively, which may need to recruit different cognitive and neural processes during speech perception depending on the listeners’ age. Specifically, it has been suggested that the downstream brain areas (e.g., bilateral inferior frontal regions) are involved in the processing of external distortions, whereas the upstream areas (e.g., left temporal regions) are responsible for the processing of internal distortions (Adank et al., 2012). The age-related differences in the use of semantic context to assist the recognition of different kind of distorted speech might be associated with the developmental/aging changes in cortical/subcortical networks that process the target speech signal in the presence of the two kinds of signal degradation, which calls for further investigation in future studies.

The masker stimuli used in the current study are consonant-misplaced sentences that are syntactically anomalous and semantically incomprehensible, and thus primarily pose energetic (as opposed to informational) masking on the targets. One concern might arise with the specific masking effects on the current findings. Various kinds of maskers (e.g., broadband noise, one- and multi-talker babble) have different acoustic/phonetic properties and thus differ greatly in masking targets and distracting listeners. Further studies using different kinds of interfering sounds are necessary to clarify how children’s speech recognition is affected by characteristics of maskers.

## Conclusion

Our current results provide supporting evidence that children use semantic context to assist speech recognition in quiet and adverse listening backgrounds. Although the abilities to recognize speech in adverse listening backgrounds and to use semantic context to assist speech recognition appear to have well developed in normal children aged 9 and beyond, younger children depend more on natural *F*_0_ contours in speech recognition than older children. These developmental changes in the influences of semantic context and *F*_0_ contours might reflect the differences in auditory and cognitive resources associated with processing of the two types of information in speech perception.

## Author Contributions

Research design: HZ, YL, CQG, LZ, and HS. Data collection: HZ, ML, and YL. Data analysis: HZ, YL, and ML. Manuscript preparation: LZ, YZ, and HS.

## Conflict of Interest Statement

The authors declare that the research was conducted in the absence of any commercial or financial relationships that could be construed as a potential conflict of interest.
